# Determinants of unintended pregnancies in rural Ghana

**DOI:** 10.1186/1471-2393-14-261

**Published:** 2014-08-08

**Authors:** Sebastian Eliason, Frank Baiden, Barbara A Yankey, Kofi Awusabo–Asare

**Affiliations:** Department of Community Medicine, University of Cape Coast, Cape Coast, Ghana; Centre for Health Research and Implementation Support, Accra, Ghana; Georgia State University, Atlanta, USA; Department of Population and Health, University of Cape Coast, Cape Coast, Ghana

**Keywords:** Unintended pregnancy, Family planning, Parity, Contraceptive methods

## Abstract

**Background:**

Unintended pregnancies may carry serious consequences for women and their families, including the possibility of unsafe abortion, delayed prenatal care, poor maternal mental health and poor child health outcomes. Although between 1993 and 2008, unintended births decreased from 42% to 37% in Ghana, the rate of decline is low, whilst levels are still very high. This raises the need to understand factors associated with unintended pregnancies, especially among women in rural settings where the rates and risks are highest to help improve maternal health.

**Method:**

We collected data from 1,914 pregnant women attending antenatal clinic between January 2012 and April 2012 in four health facilities in the Mfantseman Municipal of the Central Region of Ghana. We used bivariate and multivariate logistic regression analyses to explore how socio-demographic characteristics, past reproductive health experiences, partner characteristics and relations, awareness and past experience with contraceptives, influenced the status of women’s current pregnancy (whether intended or unintended).

**Results:**

The mean age of the 1,914 respondents in this study was 25.6 ± 6.5 years. Seventy percent (70%) said the pregnancies they were carrying were unintended. The odds of carrying unintended pregnancy among women with five or more children were higher than those with one to two children [AOR 6.06, 95% CI (3.24-11.38) versus AOR 1.48, 95% CI (1.14-1.93)]. Women with other marital arrangements showed significantly higher odds of carrying unintended pregnancy compared to those married by ordinance (Muslim or Christian wedding). Women not living with their partners exhibited increased odds of having unintended pregnancies compared to women who lived with their partners (AOR 1.72, 95% CI: 1.28 - 2.30). Awareness of traditional methods of family planning (withdrawal and rhythm) was associated with lower odds of having unintended pregnancy compared to non-awareness (AOR 0.66, 95% CI (0.49-0.89).

**Conclusions:**

In this study, important risk factors associated with unintended pregnancies were: parity, living arrangements with partner, marriage by ordinance and awareness of traditional, non-pharmacological contraceptive methods. Family planning interventions targeting different groups of women, especially during the postpartum period, would be essential to reduce rates of unintended pregnancies and promote positive health outcomes.

## Background

Unintended pregnancies refer to pregnancies that are not wanted or those that are mistimed at the time of conception [1]. Out of the 208 million pregnancies estimated worldwide, in 2008, 41% were unintended [2]. Rates of unintended pregnancies though declining world-wide are still high. Rates of unintended pregnancies declined by 20% from 71 to 57 per 1000 from 1995 to 2008 among women aged 15 to 44 years in Low- and Middle-Income Countries (LMICs) [3]. In 2008, 75 million women in LMICs reported that their pregnancies were unintended [4] with 23% of these pregnancies occurring in Sub-Saharan Africa [5]. Guttmacher Institute and the United Nations Population Fund (UNFPA) estimated the level of unintended pregnancies in 2008 at 49 per 1000 pregnancies in Asia, 72 per 1000 in Latin America and the Caribbean and for women aged 15 – 44 years in Africa, 86 per 1000; that of Africa was rated as the highest [6] and stated that in Ghana, 37 percent of all births are unintended [7].

Unintended pregnancies may carry serious consequences for women and their families, including possible unsafe abortion, delayed prenatal care, poor maternal mental health, reduced mother/child relationship quality, poor developmental outcomes for children, physical abuse and violence against women, increased risk of low birth weight of babies as well as increased maternal morbidity and mortality [2, 8, 9]. Available data suggests that induced abortion and related complications are the most common outcomes of unintended pregnancies [10]. It is estimated that in Ghana, induced abortions account for about 12% of maternal deaths, third after hemorrhage (22%) and unclassified causes (14%) [10]; furthermore, the proportion of unintended births also showed a decreasing trend: From 1993 to 2008, unintended births decreased from 42% to 37% [11]. In-spite of this decrease, the rate continues to be high and is estimated to be about 0.7 per woman [12, 13].

The high rate of unintended pregnancies in Sub-Saharan Africa, including Ghana, attests to poor access to reproductive health care especially family planning, inadequate reproductive health rights and low empowerment of women. Partly due to these prevailing situation, Ghana and most Sub-Saharan African countries are not likely to attain Millennium Development Goals (MDGs) 3, 4 and 5. Targeted interventions, especially during the postpartum period when an unintended pregnancy can be of great risk to mother and baby [14] would be essential, if the rates of unintended pregnancies are to be reduced, to promote positive health outcomes. To achieve this objective, factors that are associated with unintended pregnancies need to be investigated and understood. Studies conducted in the United States, Asia, Middle East and Latin America have revealed several demographic and socio-economic factors as predictors of unintended pregnancies; among them are contraceptive failure, lack of access to contraception, religious beliefs and poor knowledge on contraception, fertility and pregnancy, a history of previous unintended pregnancy, insufficient reproductive health education, desire for at least two children, parity of five, lack of communication or support within the relationship, husband’s reluctance to limit family size, and sexual violence [15–18].

In Ghana, where the situation is critical, very few studies have been undertaken on unintended pregnancies. For instance, one study [12] only detailed analysis of the predictors of unintended pregnancies. Some are listed as age, marital status, abode, educational status, profession, gravidity and parity, poverty or inadequate resources for raising a child, stigma against unmarried mothers, a cultural preference for sons, completion of family size, disagreement between spouses about family size, poor access to family planning services, and poor understanding of risks associated with unintended pregnancy [3, 12]. The objective of this study is to contribute to the search for predictors of unintended pregnancies in Ghana through a survey among pregnant women attending antenatal clinics in rural and semi-urban health facilities in the Mfantseman Municipal of the Central Region.

## Methods

This study was part of a bigger study on the factors influencing the intention of women in rural Ghana to adopt post-partum family planning (PPFP). The method has been described in an earlier publication [19]. The study was conducted at four health facilities in the Mfantseman Municipal of the Central Region. The area was selected in the Central Region because of consistent reports of adverse maternal health/family planning (FP) outcomes: It reported the highest level of teenage pregnancy (13.6% of all pregnancies in 2010), high incidence of induced abortions and low level of unmet need [11, 20].

For this study, all pregnant Ghanaian women living in the municipal and attending antenatal clinic at the Saltpond Government Hospital, Mankessim Health Centre (located in semi-urban settings - i.e.-demographically urban with population of about 42,000, economically agro-based with values, attitudes, tastes and behaviours characteristic of both urban and rural settings) and the Biriwa and Anomabo Health Centers (both rural) between January 2012 and April 2012 were targeted for interview within the premises of the health facilities, using a five-page questionnaire. Questions related to socio-demographic background, socio-demographic characteristics of male partners, issues pertaining to the nature of relationships between the respondents and the male partners, respondents’ reproductive history including status of current pregnancy (whether wanted, unwanted or mistimed), awareness and ever use of various Family Planning (FP) methods and the intention to use FP after delivery.

A total of 1900 pregnant women were targeted to be interviewed on the assumption that 50% of them will have the intention to adopt PPFP. With 80% power, it was possible to estimate the proportion of women willing to adopt PPFP within a margin of error of 3%. The municipal recorded about 4000 deliveries in 2009.

Cleaned data were exported into STATA/IC (version 11.2) for analyses. Descriptive and bivariate logistic regression analyses were conducted under the various sub-themes of the questionnaire. Two models were used in the bivariate logistic regression analyses. In Model I, tests of association were conducted between eighteen (18) independent variables and the outcome variable (unintended pregnancy). Fifteen (15) of the independent variables were found to be significantly associated (P < 0.05) with the outcome variable; however, since P < 0.05 is not appropriately robust to determine which associations were real and which were by chance, given so many tests, a second model (Model II) was introduced.

In Model II, the significance level threshold was set higher to 0.003 by conducting a Bonferroni correction. Factors found to be significantly associated with the main outcome of interest, were included in a multivariate logistic regression model (Model III), to identify significant independent predictors of unintended pregnancy. Tests of covariance were conducted among all the significant variables and those found to show covariance were dropped from model III.

The outcome variable (unintended pregnancy) was defined as any pregnancy that was not wanted at all at the time it occurred or in the future, or mistimed i.e. wanted at a later time but not at the time it occurred. An intended pregnancy was defined as any pregnancy that was wanted at the time it occurred or wanted at an earlier time but occurred later.

Ethical approval was obtained from the Ethics Review Committee of the Ghana Health Service (GHS). Institutional approval was also obtained from the Municipal Health Directorate (MHD) and the heads of the facilities where the survey was conducted. Written informed consent was obtained from each participant before the administration of questionnaires.

## Results

### Background characteristics of respondents and their relation to overall pregnancy status

A total of 1,914 pregnant women were interviewed. The mean age of these women was 25.6 ± 6.5 years, with the majority (29.7%) aged between 20–24 years. Majority (70%) indicated that the pregnancies they were carrying were unintended (mistimed 39%, unwanted 31%). There were more unintended pregnancies reported among younger (90%) than older women (80%), (P < 0.001).There was a trend towards reduced unintended pregnancies with increasing level of education. Prevalence of unintended pregnancies was high among all religious groups with the highest being among the traditionalists (82%). Prevalence of intended pregnancies was relatively higher amongst the Muslims (43%) and Catholics (36%), (P < 0.001). Expectant mothers with five or more children had high prevalence (61%) of unwanted pregnancies compared to those with up to four children. Of the 256 respondents who were single, only a tenth of the pregnancies were intended, in contrast to those who were married. A third of those who were married traditionally, engaged or cohabiting had intended pregnancies. The prevalence of unintended pregnancies among students (n = 124) was noticeably high (90%) compared to those who were employed in the formal sector as civil/public servants (32% of the 84 respondents). Of those in the informal sector (petty traders, fishmongers and farmers), three out of every four pregnancies were unintended. Intended pregnancies amongst those living in the two semi-urban settlements (Mankessim and Saltpond) were higher than those in the rural areas (Biriwa and Anomabo) (35% versus 20%), (P < 0.001) (Table 1).

**Table 1:** **Background characteristics by pregnancy status**

			Overall pregnancy status (%)			
Demographic characteristics	Sample size	Percent of total sample size (%)	Intended	Mistimed	Unwanted	Unintended*
Age**						
15-19	340	17.8	9.2	32.3	58.5	90.8
20-24	569	29.7	31.7	45.0	23.4	68.3
25-29	483	25.3	37.7	42.0	20.3	62.3
30-34	291	15.3	38.1	35.1	26.8	61.9
35-39	172	9.0	28.5	36.1	35.5	71.5
40+	56	2.9	19.6	25.0	55.4	80.4
TOTAL	1914	100.0	29.6	39.1	31.4	70.4
Education level**						
None	414	21.6	23.0	39.0	38.0	77.0
Primary	429	22.4	24.8	39.3	36.0	75.2
Middle/JSS	843	44.1	30.0	40.4	29.6	70.0
SSS/SHS/VOC	166	8.7	41.2	36.4	22.4	58.8
Tertiary	62	3.2	69.4	27.4	3.2	30.7
TOTAL	1,914	100.0	29.6	39.1	31.3	70.4
Religion**						
Christian	1,783	93.2	29.3	39.0	31.7	70.7
Muslim	88	4.6	39.8	36.4	23.9	60.2
Traditionalist	10	0.5	20.0	60.0	20.0	80.0
Other	33	1.7	22.6	41.9	35.5	77.4
TOTAL	1,914	100.0	29.6	39.1	31.3	70.4
Gravidity**						
1 -2	1,025	53.8	31.8	36.7	31.5	68.2
3 -4	531	27.9	30.5	46.9	22.6	69.5
5+	348	18.3	21.6	34.2	44.3	78.5
TOTAL	1,904^	100.0	29.6	39.1	31.4	70.4
Parity**						
0	673	35.2	30.9	31.1	38.0	69.1
1 -2	772	40.3	34.1	46.3	19.6	65.9
3 -4	353	18.4	23.1	42.2	34.8	76.9
5+	116	6.1	11.2	27.6	61.2	88.8
TOTAL	1,914	100.0	29.6	39.1	31.4	70.4
Marital Status**						
Married by Ordinance (Church/mosque)	236	12.4	49.6	32.6	17.8	50.4
Married (Traditional)	857	44.8	29.3	42.1	28.6	70.7
Engaged	282	14.7	38.1	40.2	21.7	61.9
Cohabitation	267	14.0	24.2	45.3	30.6	75.9
Divorced/Sep/Widowed	8	0.4	0.0	62.5	37.5	100.0
Single	256	13.4	9.6	26.7	63.8	90.4
Other	6	0.3	0.0	20.0	80.0	100.0
TOTAL	1,912^	100.0	29.6	39.1	31.3	70.4
Occupation**						
Fishmonger	318	16.6	21.5	38.6	39.9	78.5
Farmer	67	3.5	19.4	43.3	37.3	80.6
Petty trader	913	47.8	28.1	42.3	29.6	71.9
Civil/Public Servant	84	4.4	67.9	23.8	8.3	32.1
Student	124	6.5	9.8	30.9	59.4	90.2
Other	406	21.2	39.0	37.0	24.0	61.0
TOTAL	1,912^	100.0	29.6	39.1	31.3	70.4
Area of residence**						
Saltpond	422	22.2	36.3	38.9	24.9	63.7
Biriwa	231	12.1	21.2	35.9	42.9	78.8
Anomabo	324	17.0	20.1	41.1	38.9	79.9
Mankessim	567	29.8	34.0	36.5	29.5	66.0
Other	358	18.8	28.8	43.6	27.7	71.2
TOTAL	1,902^	100.0	29.6	39.1	31.3	70.4
Religious Denomination**						
Catholic	199	11.2	35.68	32.16	32.16	64.3
Protestant/Charis/pent	1,361	76.9	30.05	39.75	30.2	70.0
Muslim	81	4.6	43.21	39.51	17.28	56.8
Traditionalist	74	4.2	17.57	48.65	33.78	82.4
No/other religion	54	3.1	22.22	46.3	31.48	77.8
TOTAL	1,769^	100.0	30.53	39.46	30.02	69.5

*Unintended (mistimed + unwanted), (Pearson Chi2 Statistic - **p < 0.001), ^observed differences in total sample sizes (1914) are due to missing values.

### Bivariate logistic regression analyses of unintended pregnancies on independent variables

Unintended pregnancy (outcome variable) was regressed on each of the identified independent variables (Table 2, Model I). Only those independent variables that were found to be significantly correlated (P < 0.05) with the outcome were subjected to Bonferroni’s correction (Table 2, Model II). Women aged 20 years and above, had significantly lower odds of having unintended pregnancy (OR 0.83, 95% CI 0.77-0.89). Some factors that were found to be significantly associated with increased odds of unintended pregnancies are: not being married by ordinance (Muslim or Christian wedding) (OR 1.41, 95% CI 1.30-1.52); partner not living in the same house as the woman (OR 2.15, 95% CI 1.70 -2.72) and high parity (OR 1.20, 95% CI 1.12-1.29).

**Table 2:** **Binary logistic regression analyses: models I&II (bivariate analyses) and model III (multivariate analyses)**

	Model I		Model II	Model III	
Variables	OR (95% CI)	Unadjusted P-value	Bonferroni adjusted P-value	Adjusted Odds Ratio (AOR)	P-value
**Age (Ref: 15–19)**				NA	NA
20-24	0.83 (0.77-0.89)	<0.001	<0.001		
25-29					
30-34					
35-39					
40+					
**Educ status (Ref: none)**				NA	NA
Primary	0.98 (0.92-1.03)	0.45	NA		
Middle/JSS					
SSS/SHS/Vocational					
Tertiary					
**Ethnicity (Ref: fante)**				NA	NA
Others	1.00 (0.98-1.02)	0.89	NA		
**Religion (Ref: christian)**				NA	NA
Muslim	1.04 (0.99-1.09)	0.15	NA		
Traditionalist					
Others					
**Parity (Ref: 0)**					
1 − 2	1.20 (1.12-1.29)	<0.001		1.48 (1.14-1.93)	0.004
3 − 4				2.64 (1.88-3.71)	<0.001
5+			<0.001	6.06 (3.24-11.38)	<0.001
**Marital status (Ref: by ordinance)**					
Traditional rites	1.41 (1.30-1.52)	<0.001	<0.001	1.81 (1.33-2.45)	<0.001
Engaged, yet to be married				1.58 (1.10-2.28)	0.014
Cohabitation				2.91 (1.96-4.31)	<0.001
Single				7.32 (4.21-12.75)	<0.001
**Partner age (Ref: 15–19)**				NA	NA
20-29	0.97 (0.95-0.98)	<0.001	<0.001		
30-39					
40+					
**Partner religion (Ref: christian)**				NA	NA
Muslim	1.25 (1.06-1.47)	0.009	0.135		
Traditionalist					
Others					
**Partner has Chn. from other women (Ref: yes)**				NA	NA
No	1.33 (1.08-1.64)	0.008	0.120		
**Years of marriage/relationship (Ref: <1Yr.)**				NA	NA
1-4 yrs	1.02 (1.00-1.05)	0.019	0.285		
**Partner lives in same house as woman (Ref: yes)**					
No	2.15 (1.70-2.72)	<0.001	<0.001	1.72 (1.28-2.30)	<0.001
**Partner has other spouses (Ref: yes)**				NA	NA
No	1.47 (1.06-2.23)	0.019	0.285		
**Gravidity (Ref: 1–2)**				NA	NA
3 − 4	1.08 (1.12-1.14)	0.004	0.060		
5+					
**Previous abortions/miscarriages (Ref: yes)**				NA	NA
No	1.00 (1.00-1.0045)	0.043	0.645		
first pregnancy					
**Awareness of modern FP (Ref: no)**					
Yes	0.40 (0.25-0.62)	<0.001	<0.001	0.70 (0.42-1.17)	0.173
**Awareness of traditional FP (Ref: no)**					
Yes	0.50 (0.40-0.64)	<0.001	<0.001	0.66 (0.49-0.89)	0.007
**Ever use of modern FP (Ref: no)**				NA	NA
Yes	0.78 (0.64-0.95)	0.014	0.210		
**Ever use of traditional FP (Ref: no)**					
Yes	0.68 (0.55-0.82)	<0.001	<0.001	0.95 (0.75-1.21)	0.672

Respondents who were aware of modern and traditional family planning methods, and had ever used traditional methods showed significantly lower odds of carrying unintended pregnancy [OR 0.40, 95% CI(0.25-0.62); OR 0.50, 95% CI(0.40-0.64); OR 0.68, 95% CI(0.55-0.82) respectively]. Education was not found to be a significant factor influencing unintended pregnancies in this study.

### Multivariate logistic regression analyses

Controlling for all factors listed in Model III, increasing parity was significantly associated with increasing odds of unintended pregnancy. The odds of carrying unintended pregnancy among women with five or more children were higher than those with one to two children [AOR 6.06, 95% CI (3.24-11.38) versus AOR 1.48, 95% CI (1.14-1.93)]. Women with other marital arrangements showed significantly higher odds of carrying unintended pregnancy compared to those married by ordinance (Muslim or Christian wedding). Single women showed the highest odds of carrying unintended pregnancy [AOR 7.32, 95% CI (4.21-12.75]. Women not living with their partners exhibited increased odds of having unintended pregnancies compared to women who lived with their partners (AOR 1.72, 95% CI: 1.28 - 2.30). Awareness of traditional methods of family planning (withdrawal and rhythm) was associated with lower odds of having unintended pregnancy compared to non-awareness (AOR 0.66, 95% CI (0.49-0.89) (Table 2 Model III).

## Discussion

Factors which were identified to be significantly associated with the tendency to consider the pregnancy which women were carrying at the time of the survey to be unintended, included parity, marital arrangement, living arrangement with partner and awareness of traditional methods of contraception.

High parity was significantly associated with unintended pregnancy. The expectation was that the level of unintended pregnancy would be lower with increasing parity. The result indicating high odds of unintended pregnancies with increasing parity among women is an observation which would need further investigation despite similar findings from other studies. [12, 21, 22]. The relatively low exposure of rural women in Ghana to modern family planning [10] could partly explain this finding. Another possibility is that, couples looking for a particular gender may end up having more children than intended; there is evidence that parents wanting to balance the sex of their children will continue to give birth if all the children are of the same sex and especially if parents have a desire for a son. Chaudhuri, S, demonstrated from a study in India that the desire for sons, or not having any son, was associated with an increase in parity progression [23]. This finding supports prior research in South East Asia [24–27].

Studies have persistently demonstrated higher odds of unintended pregnancy among partners with other marital arrangements compared to married couples [28–31]. Lachance-Grzela & Genevieve Bouchard, explain that the advantage of married couples generally having favourable and healthier pregnancies than unmarried couples occurs only when the pregnancies are intended [28]. The finding that, women who reported other forms of marital arrangements had higher odds of unintended pregnancies compared to those married under the ordinance, presents an issue for further investigation within the Ghanaian context. It is possible that forms of marital arrangements could have implications for stability of marriage and therefore the possibility of planning pregnancies. The high odds of unintended pregnancies among single or unmarried women are not unexpected. This is especially so when pregnancy is considered to be a prelude to marriage or for solidifying a relationship [29]. Fear of infertility in future marital unions is a major driver behind this in some communities.

Two non-pharmacological contraceptive methods proved beneficial in preventing unintended pregnancies in this study. Women who were aware of withdrawal and rhythm as protective measures against unintended pregnancy, were less likely to have unintended pregnancies compared to those who were not aware. The socio-cultural context within the rural setting, myths and fear of side effects of modern contraceptives possibly influenced this finding*.*

As observed by Ikamari and colleagues [21] also, formal education was not significantly associated with pregnancy intendedness, contrary to expectation on this correlate. There was however, a trend towards reduced unintended pregnancy with increasing level of education (Table 1), which is consistent with other studies [12, 32]. It could be an emerging issue which would need further investigation within the Ghanaian context.

## Conclusions

This study has highlighted several factors associated with unintended pregnancy: parity, marital arrangement, living arrangement with partner and awareness of traditional, non-pharmacological contraceptive methods.

These results indicate that various categories of women would need to be targeted differently for family planning messages and services. For instance, the National Centre for Civic Education (NCCE), Ghana Health Service, religious bodies and Non-Governmental Organizations need to intensify the campaign on the importance of couples to opt for marriage by ordinance, since it has several advantages over other forms of marital arrangement. This study revealed that if partners lived together, the probability of unintended pregnancies may reduce. Marriage by ordinance may further strengthen this relationship and help to reduce unintended pregnancies. Campaigns on sex balancing aimed at encouraging parents to accept the sex of the children they have could be carried out, in order to limit the tendency to higher parity progression. This could be fairly easy given the fact that there are no obvious sex preferences in Ghana.

Family planning programmes may need to consider promotion of traditional, non-pharmacological methods alongside the modern methods, especially in rural settings, to improve overall contraceptive prevalence rates. For clients who do not want to adopt modern contraceptives despite all reassurances, the option of traditional methods should be offered them. This implies that health workers may need to be trained adequately to provide such services. Commitment from the Ghana Health Service Family Planning Programme would be required if this is to succeed.

Unintended pregnancy may be of greatest risk to mother and baby during the postpartum period. Family planning interventions, especially targeting this period, would be essential if the rates of unintended pregnancies are to be reduced, to promote positive health outcomes. In connection with this, pregnant women attending antenatal clinic (ANC) need to be targeted for family planning counseling before they deliver. Couple counseling should be actively explored by health workers as part of the routine antenatal care of each pregnant woman. It should be made a part of standard ANC protocol and health workers required to ensure adherence during facility and community based care.

### Study limitations

The threat of selection bias existed, but was highly mitigated, by ensuring that, the data collectors explained the study objectives and their implications very well to the respondents, before asking for consent. The time for the study was short; and this was imposed by limited funding and strict reporting requirements by funding agency. Some of the data collectors abandoned the study because of inadequate remuneration. New data collectors had to be trained to continue data collection. This brought about some delays in data analysis and reporting.

## Authors’ information

SE is a Public Health Physician and Lecturer in the Department of Community Medicine, university of Cape Coast, Ghana; FB is a Public Health Physician and Seniour Researcher at Centre for Health Research and Implementation Support; BY is a Pharmacist and PhD Candidate at Georgia State University, Atlanta, USA; KAA is a Professor in Population and Health at the Faculty of Social Science, University of Cape Coast, Ghana.

## Abbreviations

ANC: Antenatal clinic
AOR: Adjusted odds ratio
CI: Confidence interval
FP: Family planning
GHS: Ghana Health Service
LMIC: Low-and middle-income countries
MDG: Millennium development goals
MHD: Municipal Health Directorate
NCCE: National Commission for Civil Education
OR: Odds ratio
PPFP: Postpartum family planning
UNFPA: United Nations Population Fund.
